# The role of continuous glucose monitoring in the care of children with type 1 diabetes

**DOI:** 10.1186/1687-9856-2013-8

**Published:** 2013-03-26

**Authors:** Noelle S Larson, Jordan E Pinsker

**Affiliations:** 1 Department of Pediatrics, Division of Pediatric Endocrinology, Tripler Army Medical Center, 1 Jarrett White Road, Honolulu, HI, 96859, USA

**Keywords:** Adolescents, Children, Continuous glucose monitoring, Diabetes, Type 1 diabetes

## Abstract

Continuous glucose monitoring (CGM), while a relatively new technology, has the potential to transform care for children with type 1 diabetes. Some, but not all studies, have shown that CGM can significantly improve hemoglobin A1c levels and reduce time spent in the hypoglycemic range in children, particularly when used as part of sensor-augmented pump (SAP) therapy. Despite the publication of recent clinical practice guidelines suggesting CGM be offered to all children 8 years of age or older who are likely to benefit, and studies showing that younger children can also benefit, this technology is not yet commonly used by children with type 1 diabetes. Effects of CGM are enhanced when used on a near-daily basis (a use-dependent effect) and with insulin pump therapy. Therefore, coordinated strategies are needed to help children and their families initiate and continue to use this resource for diabetes care. This review introduces CGM to pediatric endocrinologists who are not yet familiar with the finer details of this technology, summarizes current data showing the benefits and limitations of CGM use in children, reviews specific case examples demonstrating when CGM can be helpful, and shows the value of both retrospective and real-time CGM. It is hoped that this information leads to discussion of this technology in pediatric endocrinology clinics as an important next step in improving the care of children with type 1 diabetes.

## Background

Continuous glucose monitoring (CGM) has been shown to be helpful in adults with diabetes and offers the potential to improve care for children with type 1 diabetes (T1D) beyond what can be achieved with self-monitoring of blood glucose (SMBG) alone. The Endocrine Society now recommends CGM use starting at 8 years of age for anyone with T1D able to use it on a near-daily basis. Still, despite frequent use in large diabetes centers, CGM is not commonly used for pediatric patients with T1D [1,2]. One reason for this is a lack of infrastructure and personnel qualified to teach patients, as access to a multidisciplinary trained team needed to teach families to use CGM effectively is generally not available to the non-academic pediatric endocrinologist [2,3]. In addition, there is concern among providers about being overwhelmed by the considerable amount of data obtained [4], and about limited reimbursement for time spent interpreting the data [5].

Previous reports show that fewer than 30% of children with T1D have a hemoglobin A1c (HbA1c) less than 8%, and children experience episodes of severe hypoglycemia more frequently than adults [6]. Accordingly, there is increasing interest among pediatric endocrinologists in using CGM to improve HbA1c levels and reduce the incidence of hypoglycemia in children. This corresponds with patient/family interest in CGM as a means to prevent hypoglycemia primarily, and improve diabetes control secondarily [7]. The full clinical potential impact of CGM, however, is far from being realized since most children with T1D do not use or have access to this resource for their diabetes care [4]. A major barrier for patients is that frequent use is required, with studies showing that both adults and children only benefit significantly if CGM is used more than 70% of the time (≥ 5 days per week) [8]. In addition, there are important limitations to CGM (such as inconsistency in both accuracy and precision when measuring low blood sugars), and clinicians who do not routinely use CGM may feel ill-equipped to educate patients properly on these limitations [9]. Identifying which children with T1D should use CGM has proven difficult and requires teaching families that CGM requires intensive management and will not replace time-consuming SMBG.

This review introduces CGM to clinicians who are not yet familiar with the details of the technology and summarizes current data showing the benefits and limitations of CGM use in children. Specific case examples are provided that demonstrate when CGM can be helpful and illustrate distinct qualities of both retrospective and real-time (RT) CGM. It is hoped that this information fosters discussion of use of this technology in pediatric endocrinology clinics that have not been routinely using this resource for the care of children with T1D.

### The current state of CGM use in children with T1D

CGM is currently available in the United States (US) in 2 forms: Retrospective and RT. The only fully retrospective CGM system on the market in the US is the iPro 2 (Medtronic Diabetes). The iPro 2 measures interstitial fluid (IF) glucose levels using a subcutaneous electrode for up to 72 hours and does not require active calibration during its use. In Europe, an additional system, the GlucoDay S (A. Menarini Diagnostics), is also approved. Both systems are referred to as “professional CGM” meaning that devices are owned by the clinic and are inserted at a clinic appointment. Data obtained by CGM is this setting is blinded and only available to view after the device and the patient’s blood glucose meter are downloaded in the clinic. The graphs and summary data generated can help patients and diabetes team members adjust their therapy. Small trials in children have shown these retrospective devices to be helpful in identifying post-prandial hyperglycemia and asymptomatic overnight hypoglycemia [10-12].

**Figure 1:**
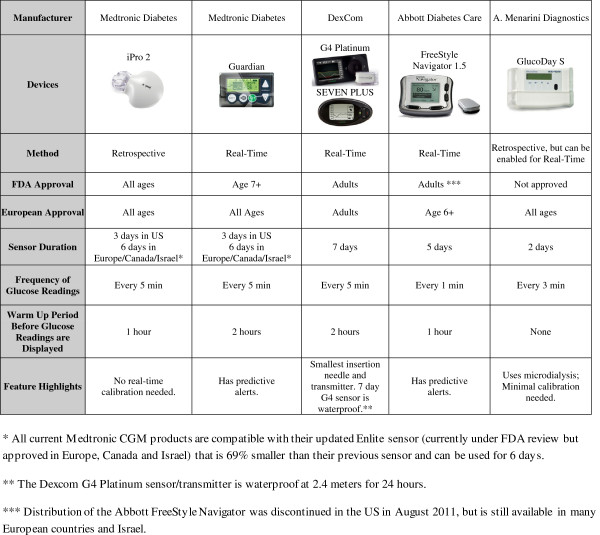
Features of currently available stand-alone CGM systems (as of March 2013).

In contrast, RT-CGM is designed for personal daily use at home, enabling patients to view their glucose levels every few minutes either on their insulin pump or on a separate receiver. Once attached by the patient at home, the device requires a short setup period followed typically by 2 or more calibrations per day. Salient features of current, commercially available stand-alone CGM systems are summarized in Figure 1.

**Figure 2:**
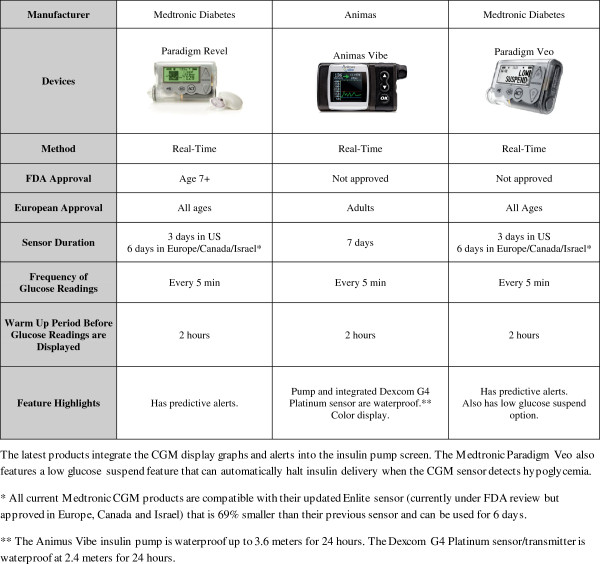
Features of currently available integrated CGM systems (as of March 2013).

All RT-CGM systems approved for use in the US (Figures 1 and 2) allow patients to download data at home for review and to either bring printouts to the clinic or submit data via the internet using bundled software packages, such as the CareLink or Diasend systems. The latest versions of all of these systems support trend graphs (viewing of glucose data in blocks of time), threshold alarms for high and low blood glucose levels, and rate of change alerts to show the direction and rate of glucose change. Some, such as the Medtronic and Abbott systems, also support glucose prediction alerts. Because all of these devices measure IF, changes in sensor readings typically lag 10 to 15 minutes behind changes in blood glucose.

Differences in blood sugar readings from the CGM and blood glucose meters, and the “false alarms” that result, can be frustrating to patients and their families [13]. The mean absolute relative difference (MARD) between sensor readings and reference glucose levels can vary by as much as 20%; such variations are especially troubling in the hypoglycemic range [14-16]. The latest generation of sensors from Medtronic (Enlite sensor – not yet FDA approved in the US) and DexCom (G4 sensor) report shorter lag times and further improvement in accuracy, especially in the hypoglycemic range [17,18]. It is important to emphasize to families that despite improvements in sensor accuracy, FDA approval for CGM devices in the US includes the recommendation that all treatment decisions (i.e., treating suspected low or high blood glucose) be based on fingerstick blood glucose, not sensor readings.

CGM devices can be used with an insulin pump as part of sensor-augmented pump (SAP) therapy or with multiple daily injections. Some systems are fully integrated into an insulin pump and use the pump display to show CGM readings (Figure 2). The Medtronic Paradigm Veo is currently the only system that includes a low glucose suspend feature that can be set to halt insulin delivery when glucose levels decline to a preset hypoglycemic threshold as determined by CGM. The sensors are approved to last only 3, 6, or 7 days, depending on the manufacturer. Thus far, off-label pediatric use of all of these systems is common.

### How can CGM help pediatric patients with T1D?

The following examples show typical blood sugar pattern problems in children with T1D, often missed by SMBG, that became apparent with CGM.

### Case 1 – detection and treatment of hyperglycemia

**Figure 3:**
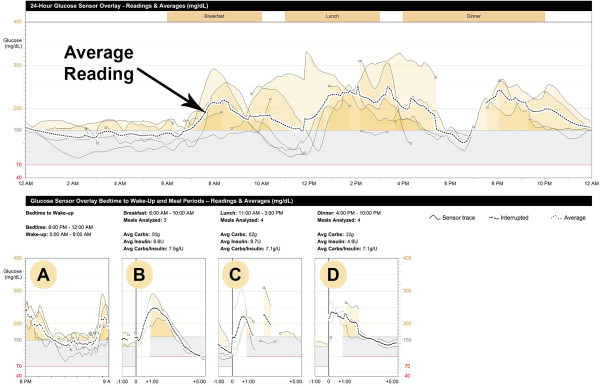
**CGM summary graph for case 1.** The entire 24-hour day appears at the top, with the dashed line showing the average reading. Specific time periods are summarized in “trend graphs.” Overnight (Panel **A**) shows an average glucose reading of 130–140 mg/dL. After breakfast (Panel **B**) shows post-prandial hyperglycemia averaging 250 mg/dL at 1 hour after the meal. Similar post-prandial hyperglycemia is shown after lunch (Panel **C**) and after dinner (Panel **D**).

Figure 3 shows the CGM summary graph of an 11-year-old female with T1D who had just started on SAP therapy. Her HbA1c was 7.4%. She was not aware of any particular problems in her blood sugar control, and download of her blood glucose meter showed most glucose readings were in her target range. Review of the CGM data summary for the few days prior to her appointment demonstrated stable overnight glucose levels (Figure 3, Summary Graph and Panel A), but examination of the CGM trend graphs revealed a pattern of post-prandial hyperglycemia (Figure 3, Panels B, C, and D). The data from CGM provided an opportunity for further improvement of glycemic control in this motivated, adherent patient and enabled her clinician to help her correlate the elevated post-prandial glucose levels with specific foods and timing of bolus insulin doses.

Can CGM use consistently improve HbA1c in children, particularly in patients, such as the one presented above, who already demonstrate good glycemic control? Meta-analyses of studies concluded prior to 2008 showed that CGM use was not superior to SMBG with regard to metabolic control among pediatric patients with T1D [19,20]. Similar results were reported from the 2008 landmark Juvenile Diabetes Research Foundation CGM randomized controlled trial, in which overall blood glucose control in children ages 8–17 years old assigned to the CGM group did not differ from the SMBG group. However, a subsequent sub-group analysis revealed that, for the 21% of children who used CGM 6+ days a week, HbA1c levels were lowered by 0.8% without an increase in hypoglycemia [21]. Subsequently, two multicenter, randomized controlled trials of adults and children with good control (HbA1c < 7.0% and 7.5%, respectively) demonstrated that RT-CGM use was associated with a mean improvement of −0.3% in HbA1c, suggesting CGM use could benefit patients whose HbA1c levels were already near their target range [22,23]. Other recent studies also support the effectiveness of frequent use of CGM in lowering HbA1c levels in children with T1D, and suggest SAP therapy may offer the most benefit to children [24-28].

### Case 2 – detection and correction of unrecognized hypoglycemia

**Figure 4:**
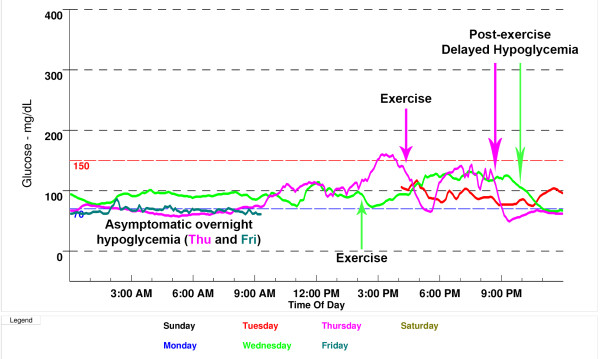
**CGM summary graph for case 2.** Asymptomatic hypoglycemia was identified overnight (Thursday and Friday night from 11 PM to 8 AM). Delayed hypoglycemia after early evening exercise was also observed, where glucose levels would rapidly drop later on between 9 and 10 PM (Wednesday and Thursday evening, arrows).

Figure 4 shows a retrospective CGM summary graph from a 14-year-old female with T1D of 2 years duration, receiving approximately 1 unit/kg/day of short acting insulin via insulin pump. She came to the clinic 3 days prior to her normal appointment and was set up with the clinic’s retrospective CGM. At the appointment her HbA1c level was found to be 7.9%, and her SMBG record revealed scattered high and low blood sugars with no consistent pattern. The patient and her family were reluctant to alter insulin doses due to a history of prior hypoglycemic seizures, and felt her current degree of blood glucose control was satisfactory. However the CGM summary graph of the previous 3 days showed asymptomatic and undetected overnight low blood sugar levels in the 60–80 mg/dL range and low blood sugar levels beginning 1–2 hours after exercise in the evening (Figure 4). Using these data from CGM, overnight basal rate on her insulin pump was decreased slightly and the patient was educated on use of the temporary basal feature (decreasing the basal rate on her insulin pump by 25% for the next 6 hours after exercise) to prevent hypoglycemia in the evening following exercise.

Fear of hypoglycemia limits many children from attaining desired HbA1c goals [29]. For many children with T1D, bedtime blood glucose checks are poor predictors of nocturnal hypoglycemia [10,11,30]. As noted in the 2012 consensus guideline for pediatric CGM, intermittent use of CGM (as demonstrated by the case history above) may detect the dawn phenomenon, postprandial hyperglycemia, asymptomatic daytime and unrecognized nocturnal hypoglycemia, and can aid in evaluating the effects of changes in treatment regimens [2]. However, initial studies of RT-CGM suggested that its use did not reduce rates of hypoglycemia [31]. The STAR1 trial of children using SAP therapy identified “failure to respond to high and low alarms, and/or appropriately dose and administer insulin,” as the main contributor to significantly higher rates of hypoglycemia [32]. The conclusion, therefore, is not that RT-CGM cannot reduce hypoglycemia in patients using insulin pumps, but rather that it needs to be used properly. More recently, multicenter, randomized controlled trials (discussed above, showing that RT-CGM use lowered HbA1c levels in adults and children with good baseline blood sugar control), demonstrated that CGM use was associated with reduced rates of hypoglycemia in both adults and children [22,23]. Further, in the last year, two industry-sponsored trials have shown significant improvements in HbA1c levels in children while also reducing time spent with blood sugar levels in the hypoglycemia range. The STAR3 trial of children using CGM showed a reduction in blood sugar level variability, favoring SAP use [25]. In addition, the SWITCH Study Group’s trial of children and adults showed that, in patients using SAP therapy, HbA1c levels, as well as time spent with blood sugar levels in the hypoglycemia range, were reduced. [28].

Greater reductions in frequency and severity of hypoglycemia are likely with further integration of CGM into the insulin pump, as now seen with the Medtronic Paradigm Veo. Retrospective analyses of children and adults using the “low glucose suspend” feature of the Paradigm Veo, which automatically suspends insulin delivery for up to 2 hours after a hypoglycemic event occurs as determined by CGM, have shown both significant reductions in exposure to hypoglycemia and prevention of profound rebound hyperglycemia after the CGM system had automatically suspended insulin delivery for up to 2 hours [33,34].

### Case 3 – glycemic variability with exercise

**Figure 5:**
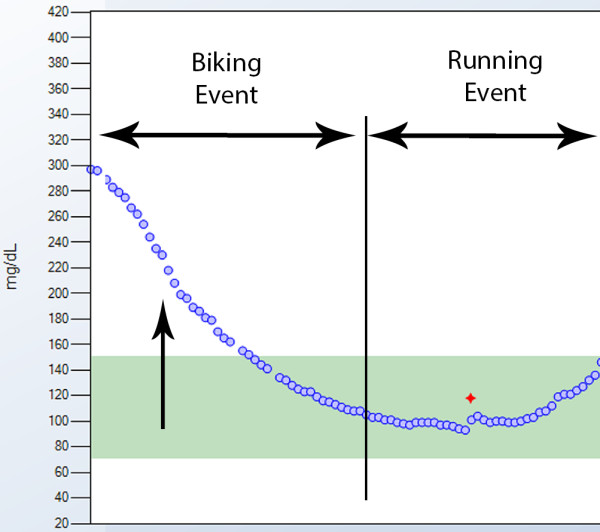
**CGM summary graph for case 3.** This 18-year-old male used RT-CGM while training for and competing in an Ironman Triathlon, 13 hours of intense physical activity. Although he was hyperglycemic at the start of the biking event, CGM showed his blood sugar began to drop rapidly (arrow). He consumed up to 75 grams of carbohydrates per hour while biking to avoid symptomatic hypoglycemia later on during the running event of the race.

The RT-CGM graph from an 18-year-old male with T1D recorded during an Ironman Triathlon is shown in Figure 5. During his training period he wore his CGM routinely, and developed a regimen of reduced basal insulin and carefully titrated carbohydrate consumption for each of the running, cycling and swimming portions of the event. The figure demonstrates how real-time awareness of a decline in sensor blood glucose levels enabled him to avoid symptomatic hypoglycemia by consuming carbohydrates at a specific rate during a part of this multi-hour competition.

CGM is a potentially powerful tool for athletes with T1D. In one study of long distance runners, CGM identified frequent hypo- and hyperglycemic episodes during and after races [35]. CGM can be applied to any of the multiple strategies used to adjust insulin for strenuous exercise, helping to customize an exercise plan for each individual patient. For example, responding to CGM “rate-of-change in blood sugar level alerts” by ingesting extra carbohydrates per a preset algorithm has been shown to help adolescents prevent exercise induced hypoglycemia [36]. Similarly, the low glucose suspend feature (described above) of the Medtronic Paradigm Veo has been shown to significantly reduce the duration and severity of exercise-induced hypoglycemia in adults, without causing significant rebound hyperglycemia [37].

## Discussion

**Figure 6:**
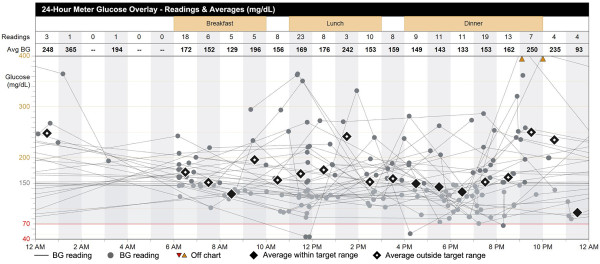
**Printout from a downloaded diabetes device**. Note that, while this 24-hour glucose overlay includes useful information, the overwhelming amount of data displayed by this graph and similar printouts may discourage physicians from analyzing them.

Continuous glucose monitoring has many theoretical and, as described above, some demonstrated virtues. However, many clinics that care for children with T1D do not have clinical or financial support to facilitate CGM use for all patients. Even today, it is not possible or practical for many clinics to routinely download standard diabetes devices, despite the clear advantages downloading offers for observation of patterns not generally seen with written log books [3]. Clinicians who are not practicing in large diabetes centers and not exposed to CGM may feel intimidated by the technology, lack sufficient time to coordinate with different online systems or download new devices in clinic, and struggle to interpret computer printouts due to format of the data displayed (Figure 6). As suggested above, the abundance of data generated and displayed can be confusing, preventing patients and clinicians from using the data well to make timely and informed decisions [4]. To avoid such problems, clinics using CGM should have a program that allows patients to: 1) download devices at home and print out specifically desired summary graphs, pie charts, etc. or 2) download devices upon arrival in clinic, with a Certified Diabetes Technologist Clinician assigned to obtain and prepare the relevant data, or 3) download devices online [38,39].

As with any technological aid for diabetes, the major issue with CGM is encouraging consistent use [40]. While early acceptance of CGM is predictive of extended use, [41] children in particular show waning adherence over time. It is important for families to understand at the outset of CGM use that this technology will not reduce the need to invest effort in diabetes management. In fact, use of RT-CGM is often more time consuming for patients because it forces them to constantly focus on diabetes care [13,42]. Consequently, it is important to offer achievable plans with realistic expectations for patients so they can experience some successes with the technology from the start. Targeting of CGM to patients and families can also be enhanced by offering in-clinic use of retrospective CGM to allow patients to try CGM without fully committing to it. Those who find it helpful can then be offered RT-CGM for personal use.

It is also important for clinics themselves to have realistic objectives regarding CGM implementation. Larger centers with “diabetes technology clinics” typically start only a few children per month on CGM due to the time and resources needed for training [13]. Some clinics offer classes that introduce the concept of CGM, provide hands-on exposure to the equipment, and then prescribe CGM for patients and families expressing interest and motivation. Use of standard reference materials (e.g. “Understanding Insulin Pumps and Continuous Glucose Monitors” [43]) is recommended as a curriculum to teach families to advance from insulin pump to SAP therapy. Instruction of families in the use of evidence-based algorithms to adjust their insulin regimen can also be extremely helpful. One model is to use the rate of change of glucose levels to alter the pre-meal bolus, with a change of 1–2 mg/dL/min indicating the need for a 10% adjustment in bolus dosing (up or down), and adjusting the bolus dose by 20% when the CGM system alerts that glucose levels are changing by more than 2 mg/dL/min [3,44]. (In smaller children who exhibit greater insulin sensitivity, these percentages may need downward adjustment to 5% and 10%, respectively). With this strategy, patients immediately see that CGM enables use of dynamic data to adjust their plan of care beyond what was previously offered by SMBG.

The cost of CGM remains a significant obstacle. It has been estimated that CGM sensors cost over $4,000 per person year in the US [31]. Although some insurance companies now provide reimbursement for these devices, particularly in patients with frequent hypoglycemia, coverage for CGM overall remains inconsistent. In addition, payment to providers for initiating CGM and interpreting data remains challenging. Clinicians can claim reimbursement in the US for the initiation of CGM and interpretation of CGM data using Current Procedural Terminology codes 95250 and 95251, although there are strict limitations on the use of these procedure codes amongst different insurers [45]. Other countries have different fee structures, with some European countries now routinely covering retrospective CGM up to 4 times per year [5,46].

Many additional challenges remain before CGM can be offered routinely to all children. While patient and family motivation predicts continued use [32], even the most motivated families often report using CGM only intermittently, despite recommendations for continuous wear, due to inability to tolerate the large size of the sensors and transmitters. Proper orientation regarding differences between devices and the advantages and disadvantages of each system is therefore very important (Figures 1 and 2). Even after all of the data has been presented, there are occasions where CGM identifies erratic glycemic control without an obvious pattern. When this occurs in patients who are already compliant with their care plan, it may not be clear as to what intervention will fix the underlying problem. These issues can potentially discourage clinics from supporting CGM in any form. Other challenges include finding the right combination of adhesive and skin site preparation with sensors sometimes falling off early or causing excessive skin irritation [13,47], and “nuisance alarms” that interfere with daily activities. These alarms may go off in the middle of the night for low or high blood sugars and may not correlate with blood glucose meter readings or be set at appropriate thresholds [42]. Because there can be a very large error rate for modern CGM systems at extreme but clinically relevant glucose concentrations, particularly for glucose values < 70 mg/dL when the glucose is changing rapidly [9], patients and families must be made aware of the limitations of the device they are using.

Frequent problems encountered by children and adolescents using CGM and possible solutions to these problems are listed in Table 1. These problems likely contribute to decreased use of CGM and negative results reported in some trials of younger children [48]. To address these barriers, details on site insertion, taping techniques, and strategies for starting CGM have been published by groups with clinical experience in this field, and should be read and understood by clinic support staff [13]. Using these techniques, many smaller children can be taught to tolerate CGM well, and for those young children already on insulin pump therapy, SAP therapy has been shown to be effective [27].

**Table 1:** Common problems seen when starting CGM in children and adolescents, and potential solutions

**Problem**	**Potential solution**
• Painful sensor insertions.	• Apply a lidocaine-based cream 45 to 60 minutes prior to insertion.
	• Apply a cool pack prior to insertion (may increase the risk of bleeding at the insertion site).
• Sensors do not adhere to the skin or cause irritation.	• Different tapes and wraps may need to be used.
	• Tegaderm, or a moleskin tape (Duoderm), can be placed under the sensor and transmitter to act as a barrier.
• Too many “nuisance alarms” that do not agree with SMBG.	• Explain the concept of “lag time” upfront.
	• Limit calibration to times when blood glucose levels are not changing rapidly.
	• Lower alarm threshold to 70 mg/dL, and use only the low glucose alarm when first starting CGM.

## Conclusions

CGM has the potential to transform care for children with T1D, particularly when used as part of SAP therapy. Consensus guidelines now exist to help physicians choose the appropriate pediatric patient for CGM use [2]. These guidelines encourage discussion with patients to provide realistic expectations, understand limitations, and target use of specific features of CGM that allow for maximizing patient benefit. Recent advances such as the low glucose suspend feature of the Medtronic Paradigm Veo show that the first step in “closing the loop,” with CGM sensors directing insulin delivery from the insulin pump, has already begun. Advancement in CGM-linked smart telemedicine systems [4,49], computerized analysis of CGM data [50], and smaller insertion needles and devices should ultimately allow pediatric endocrinologists to offer routine use of CGM to all children with T1D.

## Consent

Written informed consent was obtained from the patients for publication of this report and any accompanying images.

## Abbreviations

CGM: Continuous glucose monitoring; FDA: Food and drug administration; HbA1c: Hemoglobin A1c; IF: Interstitial fluid; MARD: Mean absolute relative difference; RT-: Real-time; SMBG: Self-monitoring of blood glucose; T1D: Type 1 diabetes; SAP: Sensor-augmented pump; US: United States

## Competing interests

This manuscript was not funded by any source. JEP has received prior grant funding for diabetes technology research from the U.S. Army Public Health Command’s Health Promotion and Prevention Initiatives (HPPI) program, as well as from the U.S. Army Medical Department’s Advanced Medical Technology Initiative (AAMTI), through the Telemedicine and Advanced Technology Research Center (TATRC). JEP also currently serves on the American Academy of Pediatrics (AAP) PREP Self-Assessment Editorial Board.

## Authors’ contributions

NSL made substantial contributions to the conception, implementation, writing, reviewing and editing of the manuscript. JEP participated in the conception, planning, implementation, writing, reviewing and editing of the manuscript, and gave final approval of the version to be published. All authors have read and reviewed the final manuscript.
